# Overexpression of cannabinoid receptor 2 is associated with human breast cancer proliferation, apoptosis, chemosensitivity and prognosis via the PI3K/Akt/mTOR signaling pathway

**DOI:** 10.1002/cam4.6037

**Published:** 2023-05-23

**Authors:** Qiang Song, Wenjin Zhang, Dan Shi, Zhiliang Zhang, Qiurong Zhao, Mengyuan Wang, Man Huang, Juanjuan Meng, Wei Cui, Xiaohe Luo

**Affiliations:** ^1^ Department of Central Laboratory Chongqing University Three Gorges Hospital Chongqing University Wanzhou, Chongqing China; ^2^ Department of Pathology, Chongqing University Three Gorges Hospital Chongqing University Wanzhou, Chongqing China; ^3^ Department of Breast Surgery Chongqing University Three Gorges Hospital, Chongqing University Wanzhou, Chongqing China

**Keywords:** apoptosis, breast cancer, CB2, PI3K/Akt/mTOR, proliferation, resistance

## Abstract

**Introduction:**

The cannabinoid receptor 2 (CB2) is mainly involved in the immune system. However, although CB2 has been reported to play an anti‐tumor function in breast cancer (BC), its specific mechanism in BC remains unclear.

**Methods:**

We examined the expression and prognostic significance of CB2 in BC tissues by qPCR, second‐generation sequencing, western blot, and immunohistochemistry. We assessed the impacts of overexpression and a specific agonist of CB2 on the growth, proliferation, apoptosis, and drug resistance of BC cells in vitro and in vivo using CCK‐8, flow cytometry, TUNEL staining, immunofluorescence, tumor xenografts, western blot, and colony formation assays.

**Results:**

CB2 expression was significantly lower in BC compared with paracancerous tissues. It was also highly expressed in benign tumors and ductal carcinoma in situ, and its expression was correlated with prognosis in BC patients. CB2 overexpression and treatment of BC cells with a CB2 agonist inhibited proliferation and promoted apoptosis, and these actions were achieved by suppressing the PI3K/Akt/mTOR signaling pathway. Moreover, CB2 expression was increased in MDA‐MB‐231 cell treated with cisplatin, doxorubicin, and docetaxel, and sensitivity to these anti‐tumor drugs was increased in BC cells overexpressing CB2.

**Conclusions:**

These findings reveal that CB2 mediates BC via the PI3K/Akt/mTOR signaling pathway. CB2 could be a novel target for the diagnosis and treatment of BC.

## Graphical abstract

It was also highly expressed in benign tumors and ductal carcinoma in situ, it may be a marker gene for breast cancer progression. CB2 could be a novel target for the diagnosis and treatment of BC

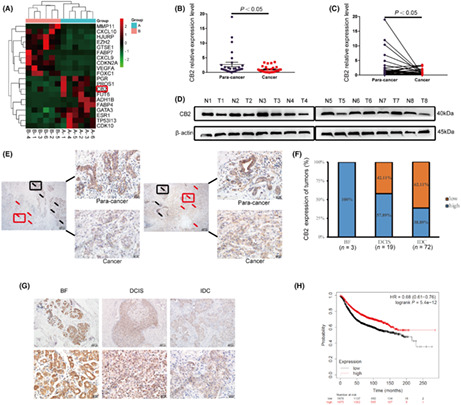

## 1 INTRODUCTION

Breast cancer (BC) is one of the most common gynecological tumors,^1^ with 2.26 million new cases in 2020, officially replacing lung cancer as the world's most common cancer.^2^ Although effective treatments for BC have improved, it still the leading cause of deaths in women.^2^, ^3^ The main treatment methods are currently radiotherapy and chemotherapy, but these are inadequate in some cases, especially in patients who develop resistance to chemotherapy drugs.^4^, ^5^, ^6^ Therefore, breast cancer for new targets is urgent.

The endocannabinoid system (ECS) is a cellular communication system that regulates a series of physiological processes in animals, including learning, memory, appetite, pain, and inflammation.^7^, ^8^, ^9^ The ECS consists of two receptors, and corresponding ligands with different related enzymes for ligand synthesis.^10^, ^11^ Its function is mainly achieved through the interactions of cannabinoids with the two G protein‐coupled receptors, cannabinoid receptor 1 (CB1), and cannabinoid receptor 2 (CB2), respectively. *CB1* and *CB2* are expressed at different sites, and are expressed in the nervous system and immune system, respectively. Increasing evidence has demonstrated an anti‐tumor function of *CB2* in many types of cancers, such as liver cancer,^12^ BC,^13^ colorectal cancer,^14^ and non‐small cell lung cancer.^15^ *CB2* and its specific agonists have shown anti‐proliferation, proapoptosis, anti‐angiogenesis, anti‐invasion, and anti‐migration effects in different tumor cells and animal models.^16^, ^17^ However, some studies have reported a tumor‐promoting function of *CB2*.^18^ Although studies found that *CB2* had anti‐tumor effects in BC, its specific mechanism in BC has not been fully explored.^8^, ^19^

In this study, we report the RNA sequencing analysis to investigate mRNA expression profiles and used immunohistochemistry analysis the *CB2* protein expression in BC tissues. Then we focused on the role of *CB2* and its clinical implications, biological effects and molecular mechanism in the breast cancer. *CB2* expression decreases in line with BC progression, and it may thus be a useful marker gene for BC. In addition, *CB2* inhibited the proliferation and promoted apoptosis of BC cells through the phosphoinositide 3‐kinase (PI3K)/Akt/mTOR signaling pathway in vivo and in vitro. Overall, our study indicate that *CB2* may be a useful molecular marker gene and a potential new therapeutic target for BC.

## 2 MATERIALS AND METHODS

### 2.1 Clinical samples

Clinical tissues were obtained from patients undergoing breast surgery of Chongqing University Three Gorges Hospital. Samples were collected from 2020 to 2022 for Basic Research. Tissues were immediately stored in −80°C. Pathological paraffin sections of BC were also obtained from the Department of Pathology. Based on the inclusion and exclusion criteria on breast cancer, our study included 139 BC patients, all clinical patient information is present in Table S1. This study was approved by the Ethics Committee of the Three Gorges Hospital Affiliated with Chongqing University (Ethics Number: 2020‐26), and all the patients provided informed consent.

### 2.2 Cell culture and drug treatment

MDA‐MB‐231 and MCF‐7 cells were obtained from the Cell Bank of the Chinese Academy of Sciences. MDA‐MB‐231 was cultured in Leibovitz's L‐15 medium (11415064, Gibco), at 37°C in room air with 10% FBS (10100147, Gibco), and MCF‐7 was cultured in MEM Medium (41090036, Gibco) at 37°C in 5% CO_2_ with 10% FBS. The CB2‐specific agonist JWH‐015^20^ was purchased from Sigma (J4252‐5MG; Sigma). The perifosine inhibits Akt phosphorylation was purchased from MedChemExpress (HY‐50909). The detailed results can be found in Supplementary Materials.

### 2.3 Inducible *CB2* transfection

The *CB2*‐overexpression lentiviral vector (GVB‐358) and CB2‐knockdown lentiviral vector (PM5.1) were purchased from Genechem. Plasmids were packaged as lentiviral vector particles, the lentiviral titers were 1 × 10^8^ TU/mL. The *CB2*‐overexpression lentiviral vector transfected into MDA‐MB‐231 and MCF‐7 cells at a 10 and 20 multiplicity of infection (MOI) value, respectively. BC cells were grown to a density of 40%, the complete medium was replaced with serum‐free culture medium, and then transfected with the *CB2*‐overexpression and empty vectors, respectively. Transfection enhancers concentrations of 5 ng/mL (Genechem) were added after transfection. The original medium was replaced by the complete medium after 12 h. After 72 h, 1 ng/mL puromycin was used to screen continuously for three generations to obtain a cell line stably expressing *CB2*. Similar methods was applied for a stable knockdown expression of *CB2* in MDA‐MB‐231 cell line.

### 2.4 qRT‐PCR

Total RNA was extracted by the phenol‐chloroform method. RNA concentration was calculated using a Nanodrop One spectrophotometer (ThermoFisher). A total of 1 μg of RNA was reverse transcribed cDNA.^21^ The qRT‐PCR experiments was finished according to the Minimum Information for Publication of Quantitative Digital PCR Experiments (MIQE).^22^ The forward and reverse *CB2* primer sequences were CTGCGCTATCCACCTTCCTA and ACAGCAAGTCCATCCCATGA, respectively. The primer housekeeping gene sequences were as follows: GAPDH primer (sense, 5′‐CAAATTCCATGGCACCGTCA‐3′; antisense, 5′‐GACTCCACGACGTACTCAGC‐3′); β‐actin (sense, 5′‐AGAGAGGCATCCTCACCCTG‐3′; antisense 5′‐GATAGCACAGCCTGGATAGCA‐3′).

### 2.5 Sequencing

Gene expression analysis was conducted via Illumina RNA sequencing (RNA‐seq). Twelve samples including six cancerous and six adjacent paracancerous tissues were used for transcriptomic sequencing. Sequencing libraries were sequenced using an Illumina HiSeq 2000 sequencer (Novogene). *CB2* expression was analyzed using the bioinformatics website (www.bioinformatics.com.cn).

### 2.6 Immunohistochemistry (IHC)

Tissue specimens were fixed with 4% formaldehyde, put in paraffin, and continuous 5–6 μm tissue slices were used for IHC. Paraffin sections were dewaxed and rehydrated and antigens were repaired under high temperature in sodium citrate (pH 8.0, Solarbio). Endogenous peroxidase activity was inhibited using 3% H_2_O_2_ and non‐specific binding was blocked using normal goat serum (5%). Anti‐CB2 polyclonal antibody (ab3561, Abcam; 1:100 dilution) was used for IHC staining, followed by horseradish peroxidase (HRP)‐conjugated anti‐IgM (SA5‐10288, Invitrogen; 1:1000 dilution) second antibody. The sections were incubated with streptavidin–HRP (Neobioscience), followed by 3,3′‐diaminobenzidine staining, counterstained with hematoxylin, and mounted with neutral gum. The IHC results were analyzed by physicians in the Pathology Department. IHC expression was graded as 0–4:0–2 indicated low expression, and grades 3 and 4 were defined as high expression (Figure S1A).

### 2.7 Western blot

The proteins of tissue and cells were extracted using Protein Extraction Reagent (78510; ThermoFisher), and quantified using BCA Protein Assay Reagent (Beyotime). The primary antibodies as follows: β‐actin (GTX109639; GenTex), CB2 (ab3561; Abcam), Cyclin A2 (ab181591; Abcam), Bcl2 (D17C4; Cell Signaling Technology [CST]), Bax (D2E11; CST), phosphorylated (p)‐Akt (D9E; CST), Akt (11E7; CST), p‐mTOR (D9C2; CST), and mTOR (7C10; CST) overnight at 4°C, then, goat anti‐rabbit secondary antibodies (A00834; Multi Sciences) was incubated for 1 h. Finally, images were exposed using an enhanced chemiluminescence kit (Chemstudio SA2, Analytik Jena).

### 2.8 Cell growth and proliferation assays

BC cells were inoculated in 96‐well plates at 30% confluence per well. After culturing for 0, 24, 48, and 72 h, 10 μL CCK‐8 solution (CK04; Dojindo) was added to each per well at incubation 1 h at 37°C, and the optical density was read at 450 nm in a microplate reader (SpectraMax, MolecularDevices).

### 2.9 Colony‐forming assay

BC Cells were plated in 6‐well plates of 2000 cells per well and cultured for 10 days, and then fixed with cell fixing solution, stained with 0.1% crystal violet for 5 min, and photographed under a microscope. The results were analyzed using ImageJ software. Experiments were repeated three times for each group.

### 2.10 Flow cytometry

Cells were plated into 6‐well plates (5 × 10^5^ cells/well) and treated with *CB2*‐overexpression lentiviral vector or agonist for 72 h. After 72 h, the cells and culture supernatants were collected. Apoptosis was then detected by flow cytometry using an APC/7‐AAD Apoptosis Detection Kit (Multi Science) and FITC/propidium iodide Apoptosis Detection Kit (Solarbio). Cell cycle profiles were generated by flow cytometry using PI staining in 488 nm and analyzed by flowJo software (Agilent Technologies).

### 2.11 TUNEL assay

Cells in logarithmic growth phase were spread on a slide and grown to 50% confluence, and then treated with *CB2*‐overexpression lentiviral vector or agonists. The cells were subjected to TUNEL staining using a TUNEL Cell Apoptosis Detection Kit (G1502; Servicebio), according to the manufacturer's instructions, and photographed under a fluorescence microscope.

### 2.12 Animal experiments

BALB/c nude mice, female, 6‐week‐old, were injected with 5 × 10^6^ MDA‐MB‐231 cells subcutaneously which were *CB2*‐overexpression lentiviruses. Tumor width and length were measured once a week for 4 weeks, and tumor volume was calculated as follows: length × width^2^/2. Then the tumors were collected for western blot and immunofluorescence. All animal procedures were carried out according to the USA National Institutes of Health Guidelines and were approved by the Animal Ethics Committee of Three Gorges Hospital Affiliated with Chongqing University.

### 2.13 Immunofluorescence

Paraffin‐embedded tumor tissues from nude mice were sectioned for immunofluorescence analysis. The tumor tissues were deparaffinized, dehydrated through graded alcohols, and treated for antigen repair. Then incubated with primary antibodies against Ki67 (D3B5; CST) overnight at 4°C, followed by FITC‐labeled anti‐rabbit secondary antibodies (GB22404; Servicebio) for 1 h. The images were captured with a microscope (BX63, Olympus).

### 2.14 Drug resistance experiments

MDA‐MB‐231 cells were treated with cisplatin (Qilu Pharmaceutical; 20 μM), the anthracycline doxorubicin (Pude Pharmaceutical; 1 μg/mL), or the taxane docetaxel (TaiJi Group; 5 μg/mL) for 48 h, respectively, and the *CB2* expression was determined by western blot. MDA‐MB‐231 cells were also transfected with *CB2*‐knockdown lentivirus and treated with cisplatin (0, 10, 20, 50 μM), doxorubicin (0, 0.2, 0.5, 1 μg/m), or docetaxel (0, 0.5, 1, 10 μg/m), and cell viability was evaluated by CCK‐8 assay.

### 2.15 Statistical analysis

Statistical analyses were conducted using GraphPad Prism 8.0 and SPSS 17.0. Data were expressed as the mean ± standard deviation. Results were compared between groups using two‐tailed Student's *t*‐tests or ANOVA tests. Kaplan–Meier survival curve analysis of BC patients was analyzed using the website: https://kmplot.com/analysis/. Differences were statistically significant at *p* < 0.05.

## 3 RESULTS

### 3.1 Decreased *CB2* expression correlated with BC prognosis

**FIGURE 1:**
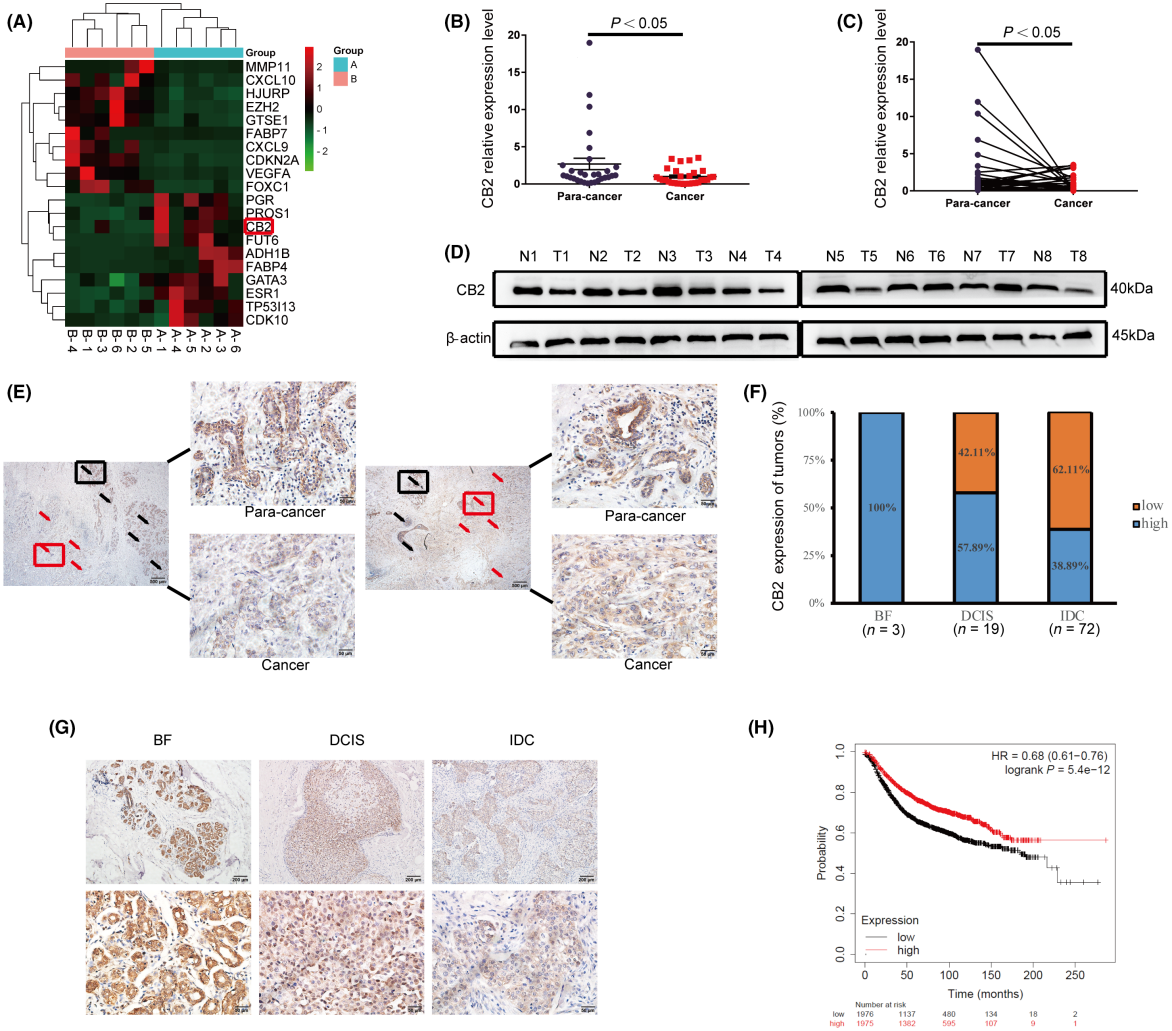
Cannabinoid receptor 2 (CB2) was downregulated in breast cancer (BC) and correlated with the prognosis of BC patients. (A) *CB2* expression was measured using RNA sequencing technologies in six paired BC samples. Group B is cancer tissue and A is adjacent tissue. (B and C) *CB2* mRNA expression level was determined in 30 paired BC and paracancerous tissues by qRT‐PCR. (D) Representative images of CB2 protein expression in eight paired BC (T) and paracancerous tissues (N). (E) Representative immunohistochemistry (IHC) images showing CB2 expression in BC and paracancerous tissues. Black arrows represent the para‐cancer tissue, red arrows represent cancer tissue. Scale bars: 500 μm, 40×; 50 μm, 400×. (F and G) Quantification and representative IHC images showing CB2 expression in 95 BC tissues. BF, breast fibroadenoma; DCIS, ductal carcinoma in situ, IDC, invasive ductal carcinoma. (H) Kaplan–Meier survival curve analysis showed that BC patients with high expression of *CB2* had longer overall survival than patients with low expression. GAPDH and β‐actin were used as normalizing genes for qRT‐PCR. Data presented as mean ± standard deviation (SD), *p < 0.05.

We investigated the association of *CB2* in the development of BC by RNA sequencing of six pairs of BC adjacent breast tissue samples. As shown by the cluster map, *CB2* expression was lower in cancerous than in tumor‐adjacent tissues (Figure 1A). Similar results to sequencing were obtained for *CB2* expression levels in 30 pairs of BC and matched paracancerous tissues by qRT‐PCR. The results confirmed that *CB2* expression was significantly reduced in BC tissues than paracancerous tissues (Figure 1B,C). Meanwhile, CB2 protein expression was lower in BC tissues (Figure 1D; Figure S1B). We then evaluated the correlations between CB2 and clinicopathological characteristics in 95 BC samples by IHC. CB2 staining was evident in 93.68% of the BC tissues (Figure S1C), and the relative intensities showed that the expression of CB2 was lower in cancer tissues compared with adjacent cancer tissues (Figure 1E; Figure S1D). In addition, benign tumors (100%, *n* = 3) and ductal carcinoma in situ (57.89%, *n* = 19) had significantly higher proportions of high‐CB2 expression compared with invasive ductal carcinoma (38.89%, *n* = 72) (Figure 1F,G; Figure S2). The correlations between clinical characteristics and CB2 expression in the 95 BC patients were listed in Table 1. According to Kaplan–Meier survival analysis, patients with high expression of *CB2* have longer overall survival than low expression (Figure 1H). Overall, these results indicated that *CB2* is involved in breast cancer progression, and a good prognostic factor in breast cancer.

**TABLE 1:** Correlation between *CB2* expression and clinicopathological features in 95 BC patients.

Characteristic	All cases	CB2		Chi‐squared	*p* value
		High	Low		
All cases	95	42	53		
Age					
<53	63	22	41	6.54	0.011*
≥53	32	20	12		
T stage					
T1	28	11	17	0.39	0.532
T2–4	67	31	36		
N stage					
N0	63	23	19	4.50	0.034*
N1–4	32	40	13		
M stage					
M0	92	41	51	0.15	0.700
M1	3	1	2		
TNM stage					
I	22	9	13	0.13	0.722
II–IV	73	33	40		

*Note*: Correlations between *CB2* expression and BC clinical features were analyzed by chi‐squared tests.

* *p* < 0.05 indicates statistical significance.

### 3.2 Overexpression of *CB2* inhibited proliferation and promoted apoptosis in BC cells

**FIGURE 2:**
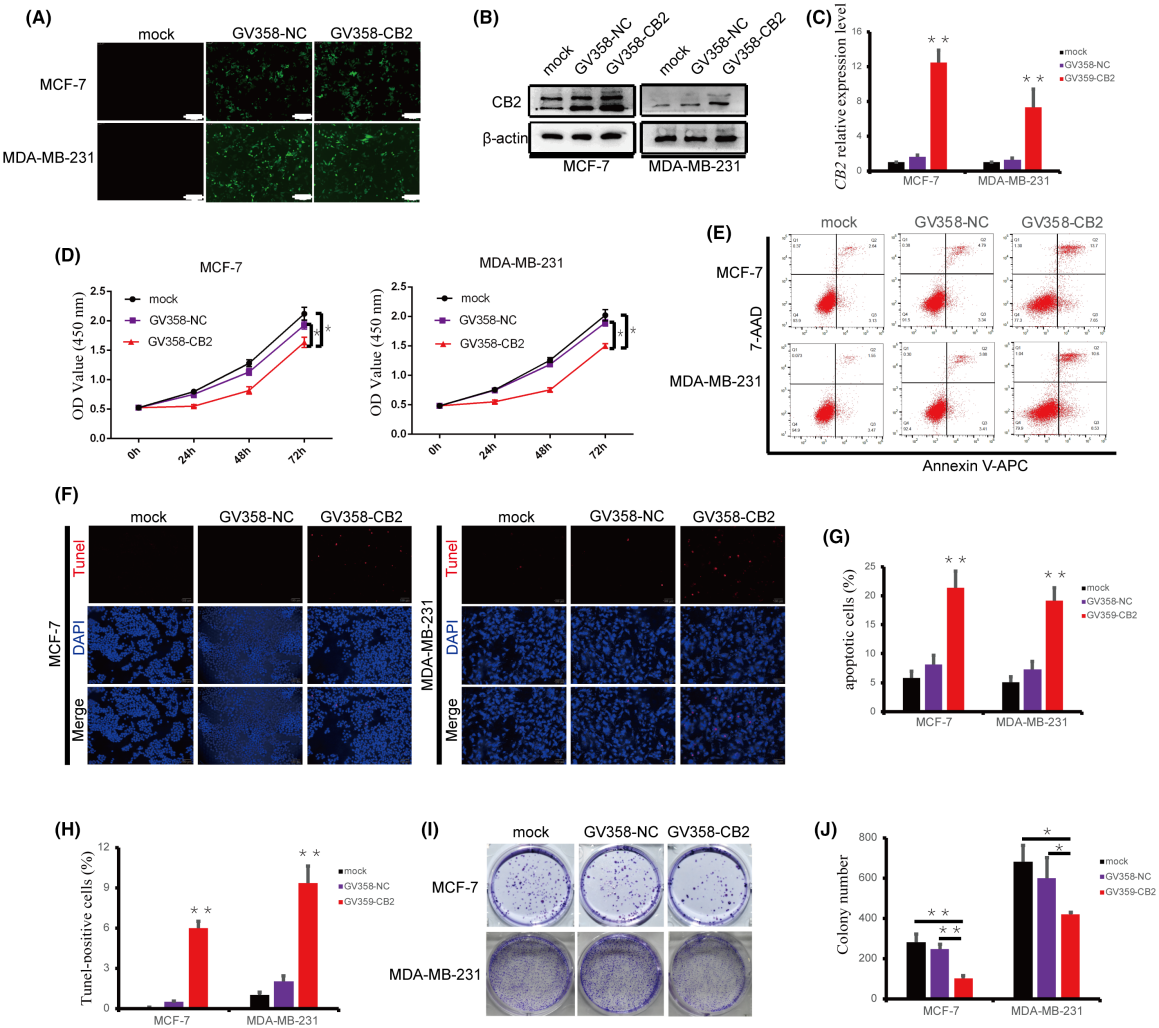
Overexpression of *CB2* inhibited proliferation and promoted apoptosis of BC cells in vitro. (A) Green fluorescence was observed in BC cells transfected with *CB2*‐overexpression plasmid. Scale bar: 200 μm, 100×. (B) *CB2* protein expression detected by western blot in BC cells transfected with *CB2*‐overexpression plasmid or control. (C) *CB2* expression was successfully upregulated by transfection with *CB2*‐overexpression plasmid, as verified by qRT‐PCR. (D) Viability of MCF‐7 and MDA‐MB‐231 cells was evaluated by CCK‐8 assay. (E and G) Apoptosis was analyzed by flow cytometry after upregulation of *CB2*. (F and H) Apoptosis ability of BC cells was detected by TUNEL assay (scale bar: 100 μm, 200×). (I and J) Survival of BC cells transfected with *CB2*‐overexpression plasmid was assessed by colony formation assay. Data presented as mean ± SD, *p < 0.05, **p < 0.01.

We explored the biological functions of *CB2* in BC cell lines by transfecting the MCF‐7 and MDA‐MB‐231 cells with *CB2*‐overexpression lentivirus plasmid (GV358‐*CB2*) for 72 h and detecting green fluorescence using a fluorescence microscope (Figure 2A). mRNA and protein expression of *CB2* are higher in cells transfected with the overexpression plasmid compared with blank control (mock) and negative control (GV358‐NC) cells, according to western blot and qRT‐PCR, respectively (Figure 2B,C). Overexpression of *CB2* markedly decreased the proliferation of BC cells by CCK‐8 assay (Figure 2D), and induced apoptosis, detected by flow cytometry using AnnexinV/7‐AAD and TUNEL staining (Figure 2E–H). Moreover, overexpression of *CB2* could decreased the S stage of MDA‐MB‐231 cells and increased the G0–G1 stage (Figure S3A). At the same time, the expression of Cyclin A2 was significantly reduced in MDA‐MB‐231 cells (Figure S3B). We further confirmed the effect of *CB2* overexpression on BC cell growth by colony formation assay. *CB2* inhibited colony formation by MCF‐7 and MDA‐MB‐231 cells compared with blank and negative controls (Figure 2I,J). These results confirmed that *CB2* inhibited the proliferation and promoted apoptosis of BC cells.

### 3.3 *CB2* agonists affected the proliferation and apoptosis of BC cells

**FIGURE 3:**
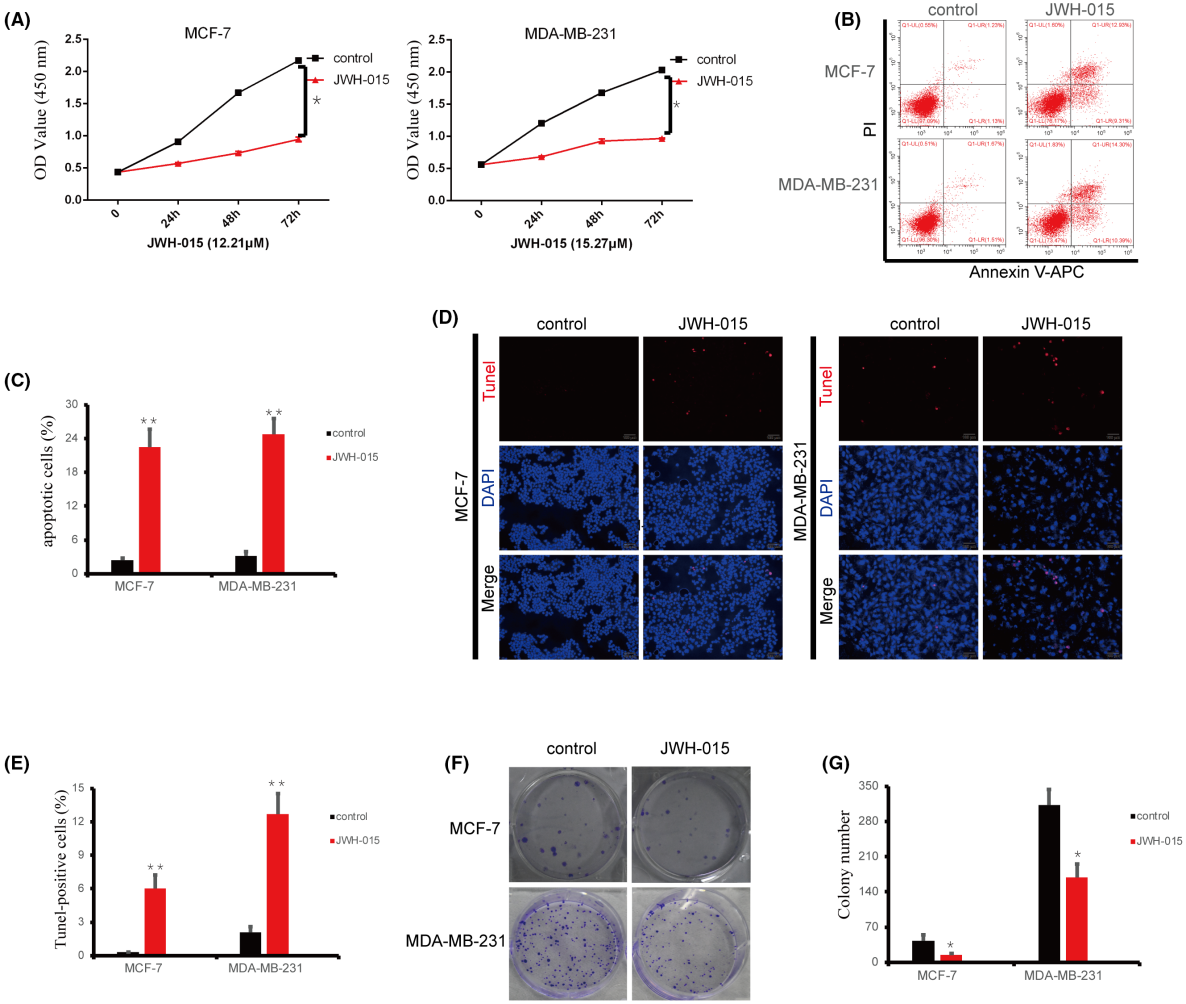
*CB2* agonists affected the proliferation and apoptosis of BC cells. (A) Viability of MCF‐7 and MDA‐MB‐231 cells treated with the *CB2* agonist JWH‐015 was determined by CCK‐8 assay. (B and C) Annexin V‐fluorescein isothiocyanate/propidium iodide staining of the MCF‐7 and MDA‐MB‐231 cells after treatment with JWH‐015. (D and E) Representative images (D) and quantification of TUNEL‐positive BC cells (E) after treatment with JWH‐015. (F and G) Growth rate increased in BC cells treated with B2 agonist. Data presented as mean ± SD, *p < 0.05, **p < 0.01.

We examined the effect of *CB2* on breast cancer cells using the specific agonist JWH‐015. BC cells were treated with various concentrations of JWH‐015 to determine the most appropriate concentration (Figure S4A,B). JWH‐015 inhibited BC cell proliferation, as shown by CCK‐8 assay (Figure 3A; Figure S4C), and promoted apoptosis of BC cell lines (Figure 3B–E). *CB2* activation also reduced the proliferation of BC cells, as shown by colony formation assays (Figure 3F,G). Collectively, these results demonstrate that a selective *CB2* agonist inhibited the proliferation and promoted apoptosis.

### 3.4 Activation of *CB2* was associated with PI3K/Akt pathway inhibition

**FIGURE 4:**
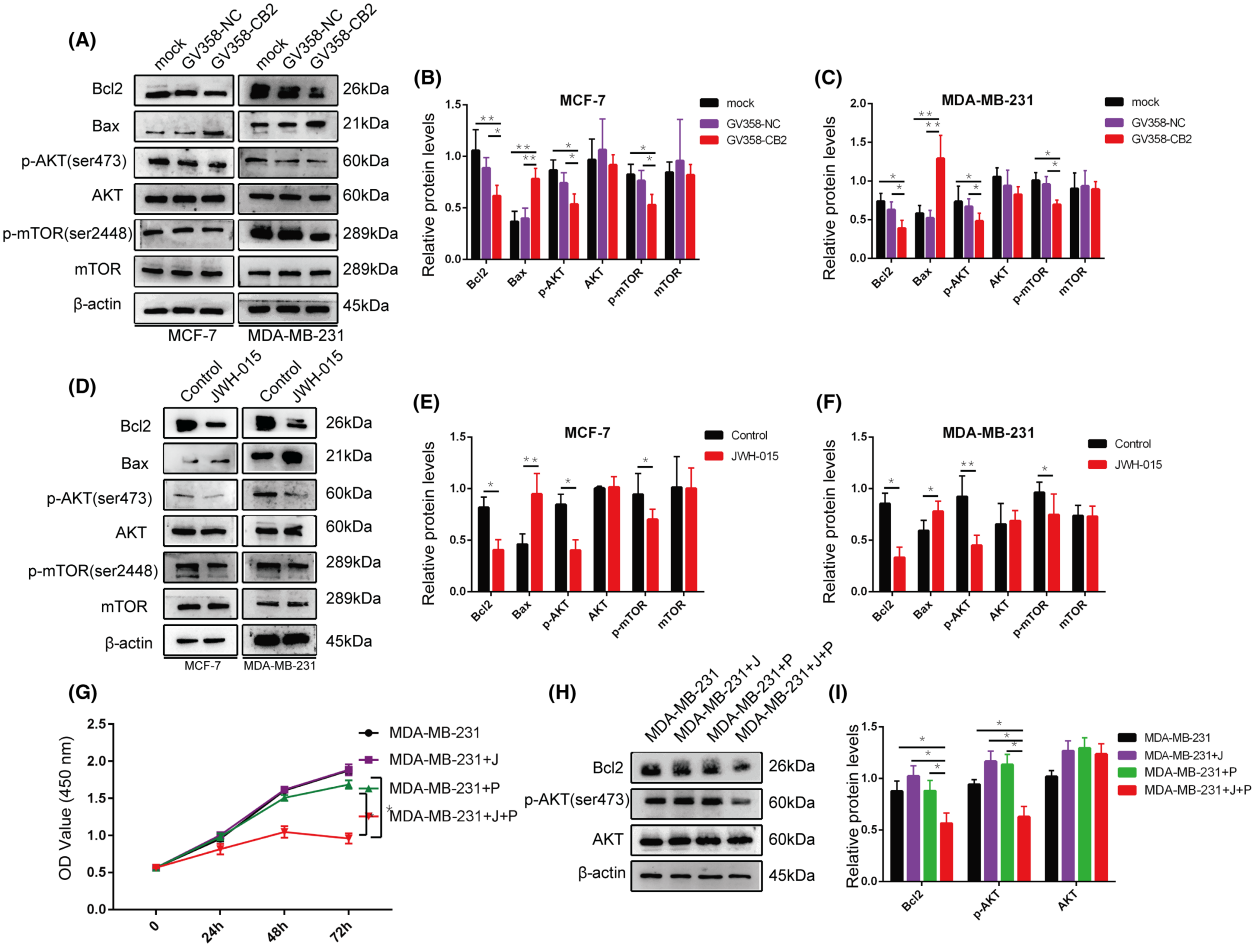
*CB2* inhibited the PI3K/Akt pathway. (A–C) western blot analysis of Bcl2, Bax, phosphorylated (p)‐Akt (p‐Akt), total Akt, p‐mTOR, and total TOR proteins in *CB2*‐overexpression MCF‐7 and MDA‐MB‐231 BC cells. (D–F) Western blotting analysis of Bcl2, Bax, p‐Akt, total Akt, p‐mTOR, and total TOR proteins in *CB2* agonist‐treated BC cells. (G) Viability of MDA‐MB‐231 cells was determined by CCK‐8 assay. Black lines: control group, purple lines: JWH‐015 group, green lines: perifosine group, red lines: JWH‐015 + perifosine group. (H and I) Western blotting analysis of Bcl2, p‐Akt, and total Akt proteins in MDA‐MB‐231 cells. Data presented as mean ± SD, *p < 0.05, **p < 0.01.

*CB2* was previously shown to either activate or inhibit the PI3K/Akt pathway by affecting the phosphorylation of Akt and mTOR.^23^ We therefore conjectured that *CB2* might affect the proliferation and apoptosis of BC cells through the PI3K/Akt signaling pathway. The expression level of anti‐apoptotic protein Bcl2 decreased and the expression level of pro‐apoptotic protein Bax increased following *CB2* overexpression in BC cells by western blot (Figure 4A–C). We evaluated the effect of *CB2* on the PI3K/Akt/mTOR by detecting the expression levels of p‐Akt, total Akt, p‐mTOR, and total mTOR. These results suggest that the expression of p‐Akt and p‐mTOR were obviously lower after *CB2* overexpression compared with the blank and negative controls, while the expression levels of Akt and mTOR were unchanged (Figure 4A–C). Similar results were obtained after treatment with the *CB2* agonist JWH‐015 in MCF‐7 and MDA‐MB‐231 cells. Bax protein expression was significantly upregulated while Bcl2, p‐Akt, and p‐mTOR levels were decreased (Figure 4D–F). We confirmed the interaction between *CB2* and the PI3K/Akt pathway using the perifosine inhibits Akt phosphorylation (1 μM) and JWH‐015 (15.27 μM) to stimulate the PI3K/Akt pathway in MDA‐MB‐231 cells. As expected, drug treatment alone had no effect on MDA‐MB‐231 cells due to the small amount of each drug. However, the combination of both drugs significantly inhibited the proliferation of BC cells (Figure 4G). Bcl2 and p‐Akt expression were significantly reduced by the combined use of the two drugs, as shown by western blot (Figure 4H,I). These results indicated that stimulation of *CB2* resulted in inhibition of the PI3K/Akt pathway.

### 3.5 *CB2* suppressed tumorigenesis of tumor xenografts in vivo

**FIGURE 5:**
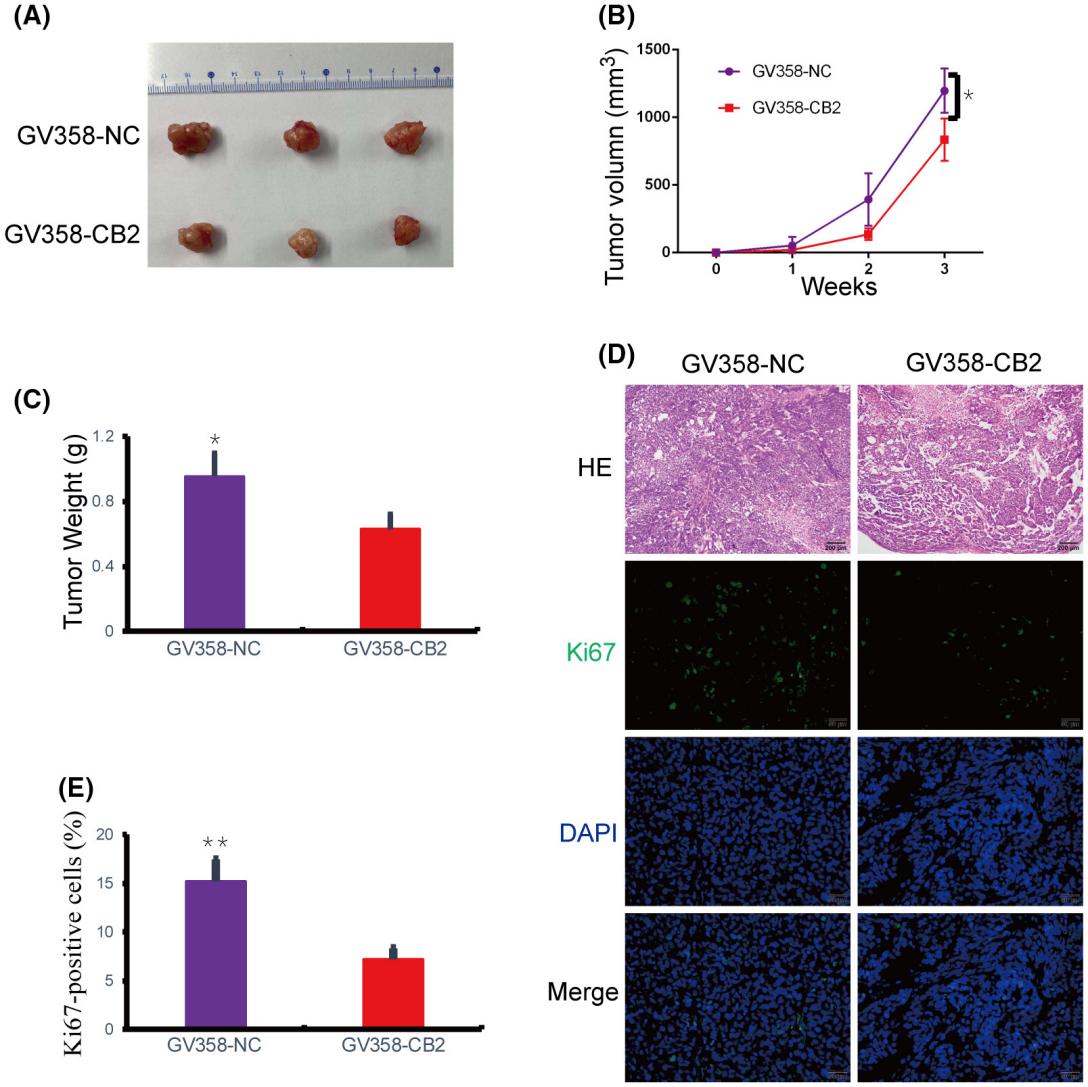
*CB2* inhibited the tumorigenesis of BC cells in vivo. (A) Representative images of tumor size are shown. (B) Tumor volumes were measured on the 1, 2, 3 week. (C) Tumor weights for each group. (D and E) HE staining and immunohistochemistry expression of Ki67 in tumors of nude mice (scale bar: 200 μm, 100×; 50 μm, 400×). Data presented as mean ± SD, *p < 0.05, **p < 0.01.

We evaluated the role of *CB2* in BC cells by establishing a stably transfected, *CB2*‐overexpression MDA‐MB‐231 cell line (triple‐negative breast cancer cell line). Tumor models were then established in female nude mice by subcutaneous injection of BC cells. Tumor volume and weight were both smaller in the *CB2*‐overexpression group compared with the control group (Figure 5A–C; Figure S4D). We also examined the morphological changes in tumor tissues isolated from nude mice with orthotopically transplanted tumors. HE staining revealed that tumors were sparser and tumor cells were reduced in the *CB2*‐overexpression group (Figure 5D). Ki‐67 IHC staining of tumor sections was used to evaluate their proliferative capacity, and Ki67 expression was significantly lower in *CB2*‐overexpressing cells (Figure 5D,E). Overall, these results demonstrated that *CB2* had tumor‐suppressive functions in BC, suggesting that *CB2* inhibited the proliferation of BC cells.

### 3.6 *CB2* enhanced the sensitivity of BC cells to anti‐tumor drugs

**FIGURE 6:**
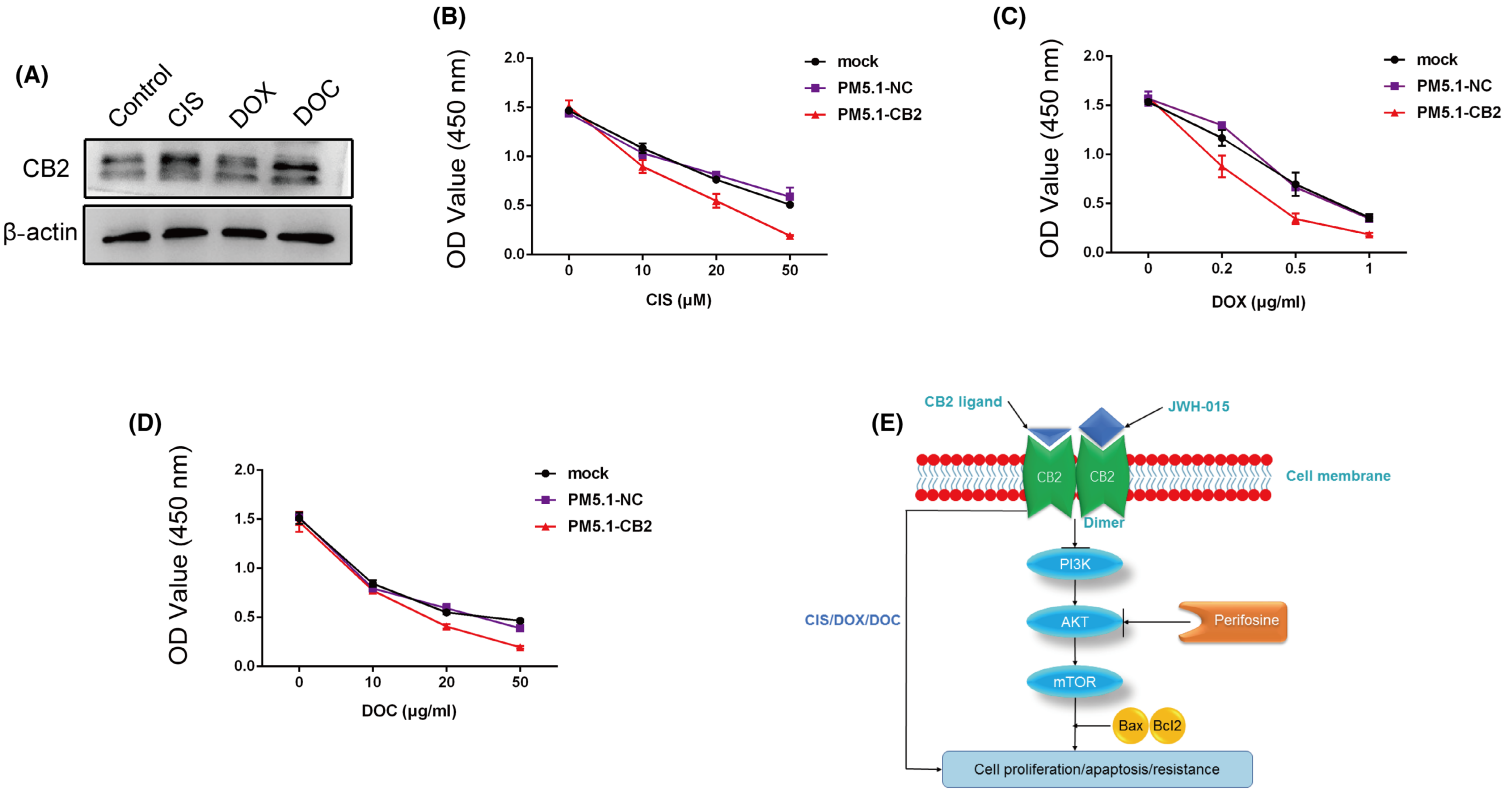
*CB2* overexpression enhanced the resistance of MDA‐MB‐231 cells to cisplatin, paclitaxel, and doxorubicin. (A) MDA‐MB‐231 cells were treated with cisplatin, paclitaxel, and doxorubicin, respectively, and CB2 protein levels were measured by western blot. (B–D) *CB2* overexpression enhanced resistance to cisplatin, paclitaxel and doxorubicin as shown by CCK‐8 assay. (E) Proposed model of the main mechanism underlying the effects of *CB2* on the regulation of BC proliferation, apoptosis, and resistance. β‐Actin levels were used as loading controls.

Cisplatin, doxorubicin, and docetaxel are commonly used as adjuvant drugs for the treatment of BC. To establish stable *CB2*‐knockdown cell line, MDA‐MB‐231 cells were transduced with lentiviral shRNA vector (Figure S4E,F). We determined the optimal concentrations of each of these drugs by CCK‐8 assays in MDA‐MB‐231 cells (Figure S4G–I). *CB2* expression increased in MDA‐MB‐231 cells following treatment with cisplatin, paclitaxel, and doxorubicin, respectively (Figure 6A). Meanwhile, stable knockdown expression of *CB2* reduced the resistance of MDA‐MB‐231 cells to cisplatin, paclitaxel, and doxorubicin (Figure 6B–D). These results indicated that knockdown of CB2 expression promoted the inhibitory effects of cisplatin, paclitaxel, and doxorubicin on the proliferation of MDA‐MB‐231 cells.

## 4 DISCUSSION

BC presents a serious danger to women's health worldwide, with the highest incidence and mortality among all female malignant tumors. The traditional treatment for breast cancer is chemotherapy, which can promote the immune system's anti‐tumor response but can also destroy the body's normal cells, resulting in undesirable side effects. The development of novel effective drugs or targets with fewer side effects is thus an urgent task in the treatment of breast cancer.^24^, ^25^ Many studies shown that the ECS is related to the development of cancer.^8^, ^10^ In the current study, *CB2* was expressed in most BC samples, with lower expression in adjacent cancerous compared with paracancerous tissues. Moreover, *CB2* was more highly expressed in benign tumors and ductal carcinoma in situ compared with invasive ductal carcinoma. This result is in disagreement with those of previous studies which highlighted the association between elevated *CB2* expression in HER2+ breast tumors and poor patient prognosis.^20^ In the present study we comprehensively investigated the involvement of the *CB2* gene in breast cancer. These results imply that *CB2* is highly expressed in poorly differentiated BC or in early‐stage BC, and may be a critical factor in its occurrence and development. Notably, high *CB2* expression was associated with better survival of patients with breast tumors. These results suggest that *CB2* is a novel oncogene in with an important role in the breast cancer.

The ECS is considered to have an anti‐tumor effect and has demonstrated efficacy in treating cancer.^26^ However, using the cannabinoid system to treat cancer is associated with mental side effects, such as dizziness, fatigue, and palpitations,^27^, ^28^ indicating the need to adopt different strategies to reduce the side effects of cannabinoids. The side effects of cannabinoids are mainly caused by the activation of CB1, suggesting that treatments should aim to selectively avoid CB1 activation and target *CB2* receptors.^29^ In the present study, we verified the anti‐tumor effect of *CB2* overexpression in the development of BC, and also verified the anti‐tumor effect of the *CB2*‐specific agonist JWH‐015 in BC. CCK‐8 and colony‐forming experiments verified that *CB2* overexpression and treatment with a *CB2*‐specific agonist inhibited the growth of BC cells, while flow cytometry and TUNEL staining showed that *CB2* promoted BC cell apoptosis. The size and weight of xenograft tumors in nude mice created using *CB2*‐overexpressing cells were significantly reduced. Expression of the proliferation‐related Ki67 was significantly reduced compared with the control group. These results suggest that *CB2* activation alone may be a potential method for avoiding neurological side effects in the treatment of BC.

The PI3K/Akt/mTOR signaling pathway is related with cell growth, translation, proliferation, translation, and metabolism.^30^, ^31^, ^32^ PI3K/Akt pathway abnormalities are common in many human cancers compared with other signaling pathways, and many components of this pathway have been implicated in the causes and effects of cancers.^33^ Interference in this pathway leads to ovarian cancer formation, cancer cell migration, and invasion, and increased resistance to chemotherapy and radiotherapy in ovarian cancer.^34^, ^35^ Alexander et al. also confirmed that cannabinoids mediated PI3K/Akt to promote apoptosis in prostate cancer.^36^, ^37^ The current results showed that *CB2* inhibited tumor growth and promoted apoptosis in BC cells, mediated via the PI3K/Akt/mTOR pathway. Overexpression or specific activation of *CB2* in BC cells reduced the expression of p‐Akt and p‐mTOR, leading to a decrease in expression of the downstream apoptosis‐related gene Bcl2 and an increase in Bax. The Akt inhibitor perifosine promoted apoptosis caused by JWH‐015 (Figure 6E). These results confirmed that *CB2* may inhibit the activation of the PI3K/Akt/mTOR pathway through a series of reactions, thereby inhibiting tumorigenesis.

Cancer drug resistance is an ongoing challenge in the treatment of many cancers.^38^ It is therefore imperative to revalidate the existing strategies used for cancer treatment and find new treatment methods based on killing of cancer cells targeted at the genetic level.^39^, ^40^, ^41^ Cisplatin, the anthracycline doxorubicin, and the taxane docetaxel are commonly used drugs for BC treatment, and are often used in combination with multiple drugs to obtain better curative effects.^42^, ^43^, ^44^ However, BC cells develop drug resistance with extended chemotherapy time, which in turn greatly reduces the effect of chemotherapy. There are currently few biomarker genes predicting drug resistance in BC.^45^, ^46^ In this study, we found that *CB2* expression increased when treated with cisplatin, doxorubicin, or docetaxel, in MDA‐MB‐231 cells, suggesting that *CB2* was involved in the chemo‐sensitivity of MDA‐MB‐231 cells to these agents. In addition, *CB2* overexpression not only had an inhibitory effect on BC cells, but also significantly improved their sensitivity to chemotherapy with cisplatin, doxorubicin, and docetaxel. This suggests that *CB2* might serve as a marker gene for drug resistance during BC treatment. However, further studies are needed to elucidate the specific mechanism of action.

## 5 CONCLUSIONS

In summary, the results of this study demonstrated that CB2 plays an important role in BC progression, and provided insights into the underlying mechanisms. Establishing the precise role played by CB2 in BC progression will not only advance our understanding of the biology of BC, but may also offer a novel therapeutic strategy via the suppression of CB2. Our results also suggest that CB2 may have a potential role as a clinical predictor of disease progression. In addition, CB2 may be related to tumor drug resistance. These results thus indicate that CB2 may be a prognostic biomarker and a promising target for the treatment of BC.

## AUTHOR CONTRIBUTIONS

**Qiang Song:** Data curation (equal); formal analysis (lead); funding acquisition (equal); methodology (equal); writing – review and editing (equal). **Wenjin Zhang:** Conceptualization (supporting); resources (equal); validation (equal); writing – original draft (supporting). **Dan Shi:** Methodology (equal); supervision (equal). **Zhiliang Zhang:** Software (equal); supervision (equal). **Qiurong Zhao:** Data curation (equal); formal analysis (equal). **Mengyuan Wang:** Methodology (equal); visualization (equal). **Man Huang:** Formal analysis (equal); supervision (equal). **Juanjuan Meng:** Resources (equal); visualization (equal). **Wei Cui:** Project administration (equal); software (equal); supervision (equal). **Xiaohe Luo:** Funding acquisition (equal); methodology (equal); project administration (equal); writing – original draft (supporting); writing – review and editing (supporting).

## FUNDING INFORMATION

This work was supported by the Natural Science Foundation Project of Chongqing, China (cstc2020jcyj‐msxmX0049; cstc2019jcyj‐msxmX0823).

## ETHICS STATEMENT

The study was conducted according to the guidelines of the Declaration of Helsinki, and approved by the ethics committee of the Three Gorges Hospital Affiliated with Chongqing University Clinical and the Laboratory Research Ethical Council (2020‐26).

## Supporting information


**Figure S1**.

Figure S2.

Figure S3.

Figure S4.

Table S1.

Supplementary Figure Captions

## Data Availability

The authors confirm that the data supporting the findings of this study are available within the article [and/or] its supplementary materials.

## References

[1] Ghoncheh M , Pournamdar Z , Salehiniya H . Incidence and mortality and epidemiology of breast cancer in the world. Asian Pac J Cancer Prev. 2016;17:43‐46. 10.7314/apjcp.2016.17.s3.43 27165206

[2] Ferlay J , Colombet M , Soerjomataram I , et al. Cancer statistics for the year 2020: an overview. Int J Cancer. 2021;149:778‐789. 10.1002/ijc.33588 33818764

[3] Rumgay H , Shield K , Charvat H , et al. Global burden of cancer in 2020 attributable to alcohol consumption: a population‐based study. Lancet Oncol. 2021;22:1071‐1080. 34270924 10.1016/S1470-2045(21)00279-5 PMC8324483

[4] Radecka B , Litwiniuk M . Breast cancer in young women. Ginekol pol. 2016;87:659‐663. 27723074 10.5603/GP.2016.0062

[5] Castaneda SA , Strasser J . Updates in the treatment of breast cancer with radiotherapy. Surg Oncol Clin N Am. 2017;26:371‐382. 28576177 10.1016/j.soc.2017.01.013

[6] Sharma S , Barry M , Gallagher DJ , Kell M , Sacchini V . An overview of triple negative breast cancer for surgical oncologists. Surg Oncol. 2015;24:276‐283. 26092709 10.1016/j.suronc.2015.06.007

[7] Wilson RI , Nicoll RA . Endocannabinoid signaling in the brain. Science. 2002;296:678‐682. 11976437 10.1126/science.1063545

[8] Dobovisek L , Krstanovic F , Borstnar S , Debeljak N . Cannabinoids and hormone receptor‐positive breast cancer treatment. Cancers (Basel). 2020;12:525. 32106399 10.3390/cancers12030525 PMC7139952

[9] McAllister SD , Soroceanu L , Desprez PY . The antitumor activity of plant‐derived non‐psychoactive cannabinoids. J Neuroimmune Pharmacol. 2015;10:255‐267. 25916739 10.1007/s11481-015-9608-y PMC4470774

[10] Bifulco M , Di Marzo V . Targeting the endocannabinoid system in cancer therapy: a call for further research. Nat Med. 2002;8:547‐550. 12042794 10.1038/nm0602-547

[11] Grimaldi C , Capasso A . The endocannabinoid system in the cancer therapy: an overview. Curr Med Chem. 2011;18:1575‐1583. 21428888 10.2174/092986711795471374

[12] Ramer R , Hinz B . Antitumorigenic targets of cannabinoids—current status and implications. Expert Opin Ther Targets. 2016;20:1219‐1235. 27070944 10.1080/14728222.2016.1177512

[13] Perez‐Gomez E , Andradas C , Blasco‐Benito S , et al. Role of cannabinoid receptor CB2 in HER2 pro‐oncogenic signaling in breast cancer. J Natl Cancer Inst. 2015;107:djv077. 25855725 10.1093/jnci/djv077

[14] Alenabi A , Malekinejad H . Cannabinoids pharmacological effects are beyond the palliative effects: CB2 cannabinoid receptor agonist induced cytotoxicity and apoptosis in human colorectal cancer cells (HT‐29). Mol Cell Biochem. 2021;476:3285‐3301. 33886060 10.1007/s11010-021-04158-6

[15] Xu S , Ma H , Bo Y , Shao M . The oncogenic role of CB2 in the progression of non‐small‐cell lung cancer. Biomed Pharmacother. 2019;117:109080. 31176172 10.1016/j.biopha.2019.109080

[16] Olea‐Herrero N , Vara D , Malagarie‐Cazenave S , Diaz‐Laviada I . Inhibition of human tumour prostate PC‐3 cell growth by cannabinoids R(+)‐Methanandamide and JWH‐015: involvement of CB2. Br J Cancer. 2009;101:940‐950. 19690545 10.1038/sj.bjc.6605248 PMC2743360

[17] Sanchez C , de Ceballos ML , Gomez del Pulgar T , et al. Inhibition of glioma growth in vivo by selective activation of the CB(2) cannabinoid receptor. Cancer Res. 2001;61:5784‐5789. 11479216

[18] Pisanti S , Picardi P , D'Alessandro A , Laezza C , Bifulco M . The endocannabinoid signaling system in cancer. Trends Pharmacol Sci. 2013;34:273‐282. 23602129 10.1016/j.tips.2013.03.003

[19] Zhang J , Zhang S , Liu Y , et al. Combined CB2 receptor agonist and photodynamic therapy synergistically inhibit tumor growth in triple negative breast cancer. Photodiagnosis Photodyn Ther. 2018;24:185‐191. 30240926 10.1016/j.pdpdt.2018.09.006 PMC6289793

[20] Aung MM , Griffin G , Huffman JW , et al. Influence of the N‐1 alkyl chain length of cannabimimetic indoles upon CB(1) and CB(2) receptor binding. Drug Alcohol Depend. 2000;60:133‐140. 10940540 10.1016/s0376-8716(99)00152-0

[21] Bashiardes S , Lovett M . cDNA detection and analysis. Curr Opin Chem Biol. 2001;5:15‐20. 11166642 10.1016/s1367-5931(00)00161-7

[22] Bustin SA , Wittwer CT . MIQE: a step toward more robust and reproducible quantitative PCR. Clin Chem. 2017;63:1537‐1538. 28606913 10.1373/clinchem.2016.268953

[23] Elbaz M , Ahirwar D , Ravi J , Nasser MW , Ganju RK . Novel role of cannabinoid receptor 2 in inhibiting EGF/EGFR and IGF‐I/IGF‐IR pathways in breast cancer. Oncotarget. 2017;8:29668‐29678. 27213582 10.18632/oncotarget.9408 PMC5444694

[24] Barzaman K , Karami J , Zarei Z , et al. Breast cancer: biology, biomarkers, and treatments. Int Immunopharmacol. 2020;84:106535. 32361569 10.1016/j.intimp.2020.106535

[25] Thorat MA , Balasubramanian R . Breast cancer prevention in high‐risk women. Best Pract Res Clin Obstet Gynaecol. 2020;65:18‐31. 31862315 10.1016/j.bpobgyn.2019.11.006

[26] Lal S , Shekher A , Puneet NAS , Abrahamse H , Gupta SC . Cannabis and its constituents for cancer: history, biogenesis, chemistry and pharmacological activities. Pharmacol Res. 2021;163:105302. 33246167 10.1016/j.phrs.2020.105302

[27] Donvito G , Nass SR , Wilkerson JL , et al. The endogenous cannabinoid system: a budding source of targets for treating inflammatory and neuropathic pain. Neuropsychopharmacology. 2018;43:52‐79. 28857069 10.1038/npp.2017.204 PMC5719110

[28] Das S , Kaul K , Mishra S , Charan M , Ganju RK . Cannabinoid signaling in cancer. Adv Exp Med Biol. 2019;1162:51‐61. 31332734 10.1007/978-3-030-21737-2_4

[29] Irrera N , Bitto A , Sant'Antonio E , Lauro R , Musolino C , Allegra A . Pros and cons of the cannabinoid system in cancer: focus on hematological malignancies. Molecules. 2021;26:3866. 34202812 10.3390/molecules26133866 PMC8270322

[30] Xu F , Na L , Li Y , Chen L . Roles of the PI3K/AKT/mTOR signalling pathways in neurodegenerative diseases and tumours. Cell Biosci. 2020;10:54. 32266056 10.1186/s13578-020-00416-0 PMC7110906

[31] Sun K , Luo J , Guo J , Yao X , Jing X , Guo F . The PI3K/AKT/mTOR signaling pathway in osteoarthritis: a narrative review. Osteoarthr Cartil. 2020;28:400‐409. 10.1016/j.joca.2020.02.027 32081707

[32] Tewari D , Patni P , Bishayee A , Sah AN , Bishayee A . Natural products targeting the PI3K‐Akt‐mTOR signaling pathway in cancer: a novel therapeutic strategy. Semin Cancer Biol. 2019;80:1‐17. 31866476 10.1016/j.semcancer.2019.12.008

[33] Shorning BY , Dass MS , Smalley MJ , Pearson HB . The PI3K‐AKT‐mTOR pathway and prostate cancer: at the crossroads of AR, MAPK, and WNT signaling. Int J Mol Sci. 2020;21:4507. 32630372 10.3390/ijms21124507 PMC7350257

[34] Ediriweera MK , Tennekoon KH , Samarakoon SR . Role of the PI3K/AKT/mTOR signaling pathway in ovarian cancer: biological and therapeutic significance. Semin Cancer Biol. 2019;59:147‐160. 31128298 10.1016/j.semcancer.2019.05.012

[35] Lau MT , Leung PC . The PI3K/Akt/mTOR signaling pathway mediates insulin‐like growth factor 1‐induced E‐cadherin down‐regulation and cell proliferation in ovarian cancer cells. Cancer Lett. 2012;326:191‐198. 22922215 10.1016/j.canlet.2012.08.016

[36] Greenhough A , Patsos HA , Williams AC , Paraskeva C . The cannabinoid delta(9)‐tetrahydrocannabinol inhibits RAS‐MAPK and PI3K‐AKT survival signalling and induces BAD‐mediated apoptosis in colorectal cancer cells. Int J Cancer. 2007;121:2172‐2180. 17583570 10.1002/ijc.22917

[37] Bossler F , Hoppe‐Seyler K , Hoppe‐Seyler F . PI3K/AKT/mTOR signaling regulates the virus/host cell crosstalk in HPV‐positive cervical cancer cells. Int J Mol Sci. 2019;20:2188. 31058807 10.3390/ijms20092188 PMC6539191

[38] Giovannetti E , Mey V , Nannizzi S , Pasqualetti G , Del Tacca M , Danesi R . Pharmacogenetics of anticancer drug sensitivity in pancreatic cancer. Mol Cancer Ther. 2006;5:1387‐1395. 16818496 10.1158/1535-7163.MCT-06-0004

[39] Panda M , Biswal BK . Cell signaling and cancer: a mechanistic insight into drug resistance. Mol Biol Rep. 2019;46:5645‐5659. 31280421 10.1007/s11033-019-04958-6

[40] Tripathi SK , Pandey K , Rengasamy KRR , Biswal BK . Recent updates on the resistance mechanisms to epidermal growth factor receptor tyrosine kinase inhibitors and resistance reversion strategies in lung cancer. Med Res Rev. 2020;40:2132‐2176. 32596830 10.1002/med.21700

[41] Longley DB , Allen WL , Johnston PG . Drug resistance, predictive markers and pharmacogenomics in colorectal cancer. Biochim Biophys Acta. 2006;1766:184‐196. 16973289 10.1016/j.bbcan.2006.08.001

[42] Liapis E , Karlas A , Klemm U , Ntziachristos V . Chemotherapeutic effects on breast tumor hemodynamics revealed by eigenspectra multispectral optoacoustic tomography (eMSOT). Theranostics. 2021;11:7813‐7828. 34335966 10.7150/thno.56173 PMC8315054

[43] Sulaiman A , McGarry S , Chambers J , et al. Targeting hypoxia sensitizes TNBC to cisplatin and promotes inhibition of both bulk and cancer stem cells. Int J Mol Sci. 2020;21:5788. 32806648 10.3390/ijms21165788 PMC7461107

[44] Bourgeois‐Daigneault MC , St‐Germain LE , Roy DG , et al. Combination of Paclitaxel and MG1 oncolytic virus as a successful strategy for breast cancer treatment. Breast Cancer Res. 2016;18:83. 27503504 10.1186/s13058-016-0744-y PMC4977613

[45] Nedeljkovic M , Damjanovic A . Mechanisms of chemotherapy resistance in triple‐negative breast cancer—how we can rise to the challenge. Cell. 2019;8:957. 10.3390/cells8090957 PMC6770896 31443516

[46] Cui W , Xiao Y , Zhang R , et al. SOHLH2 suppresses angiogenesis by downregulating HIF1alpha expression in breast cancer. Mol Cancer Res. 2021;19:1498‐1509. 34158392 10.1158/1541-7786.MCR-20-0771

