# Supplementary figures and images for: Overexpression of cannabinoid receptor 2 is associated with human breast cancer proliferation, apoptosis, chemosensitivity and prognosis via the PI3K/Akt/mTOR signaling pathway
Source: Cancer Med. 2023 May 23;12(12):13538–50. doi: 10.1002/cam4.6037 (PMC10315729; doi:10.1002/cam4.6037)

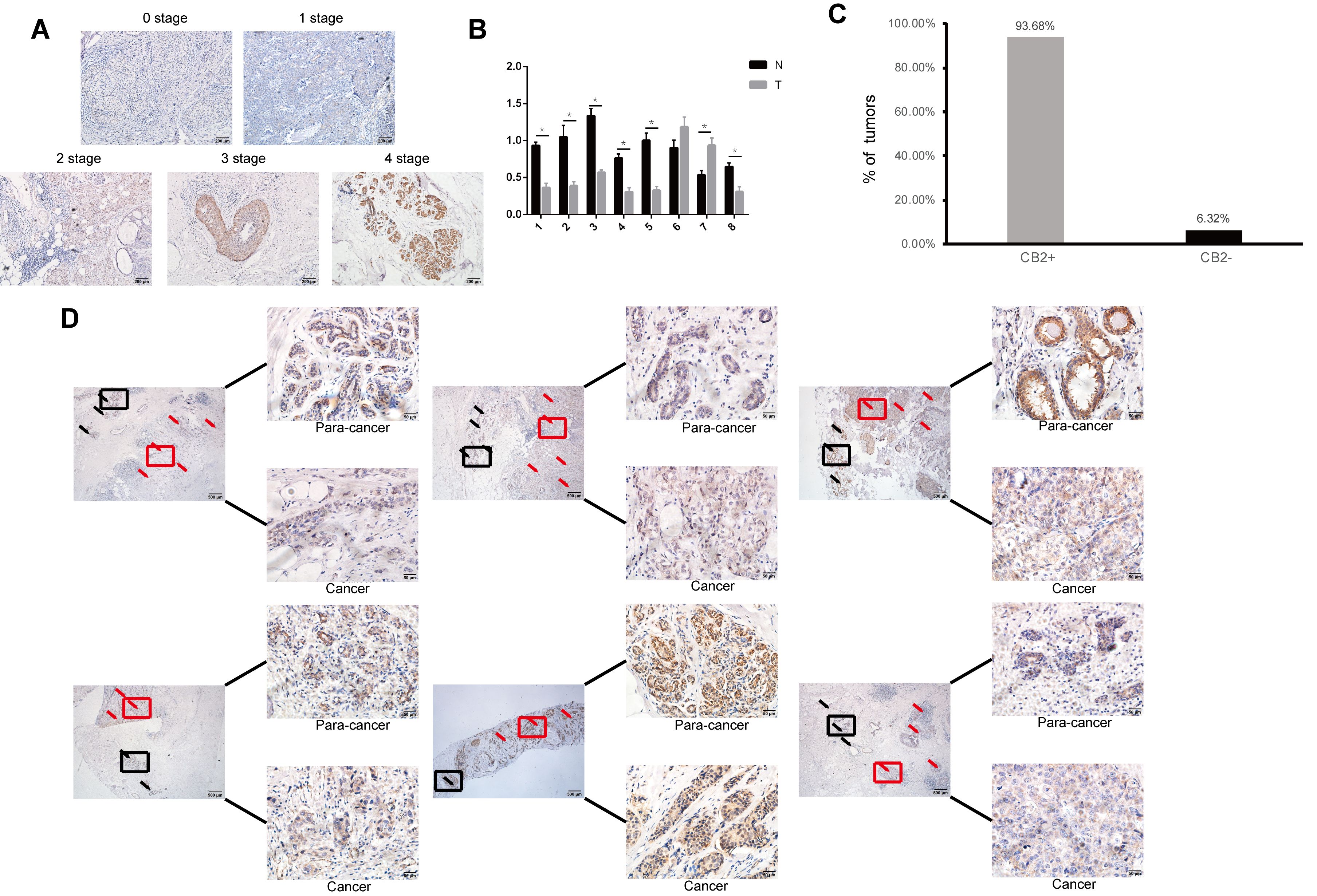

Supplement: Supplementary file 1 — Figure S1. [file CAM4-12-13538-s002.tif]

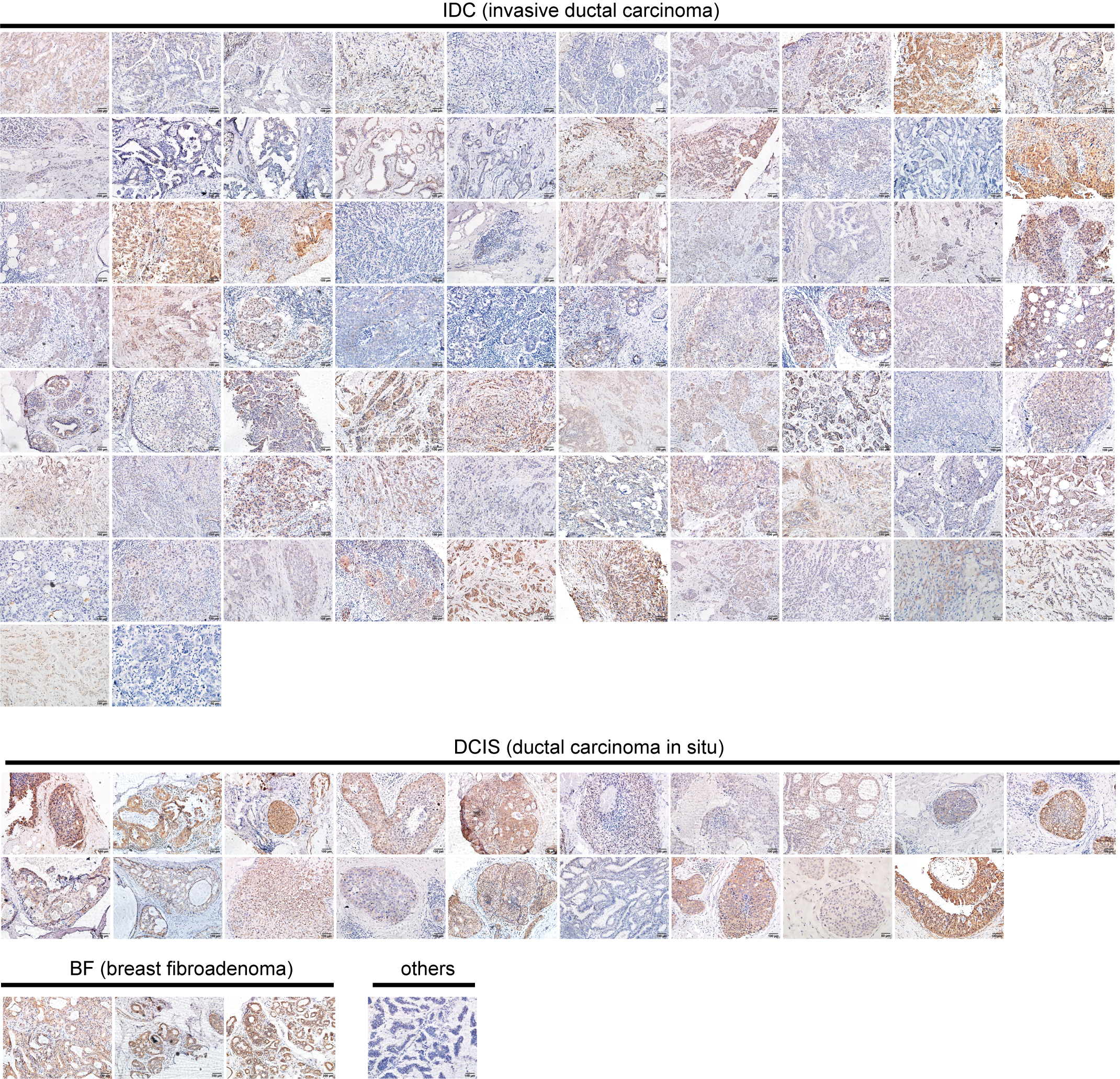

Supplement: Supplementary file 2 — Figure S2. [file CAM4-12-13538-s006.tif]

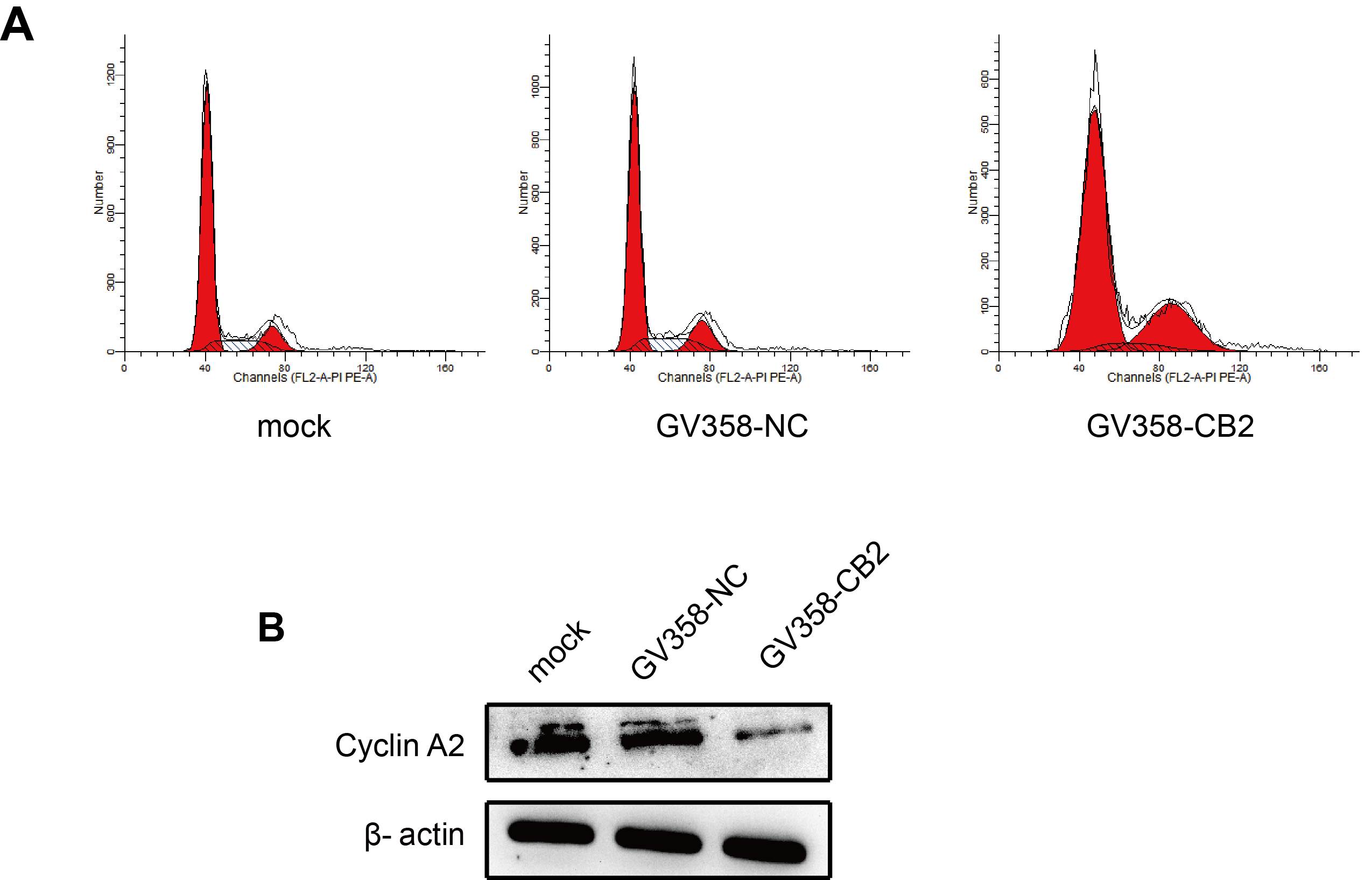

Supplement: Supplementary file 3 — Figure S3. [file CAM4-12-13538-s004.png]

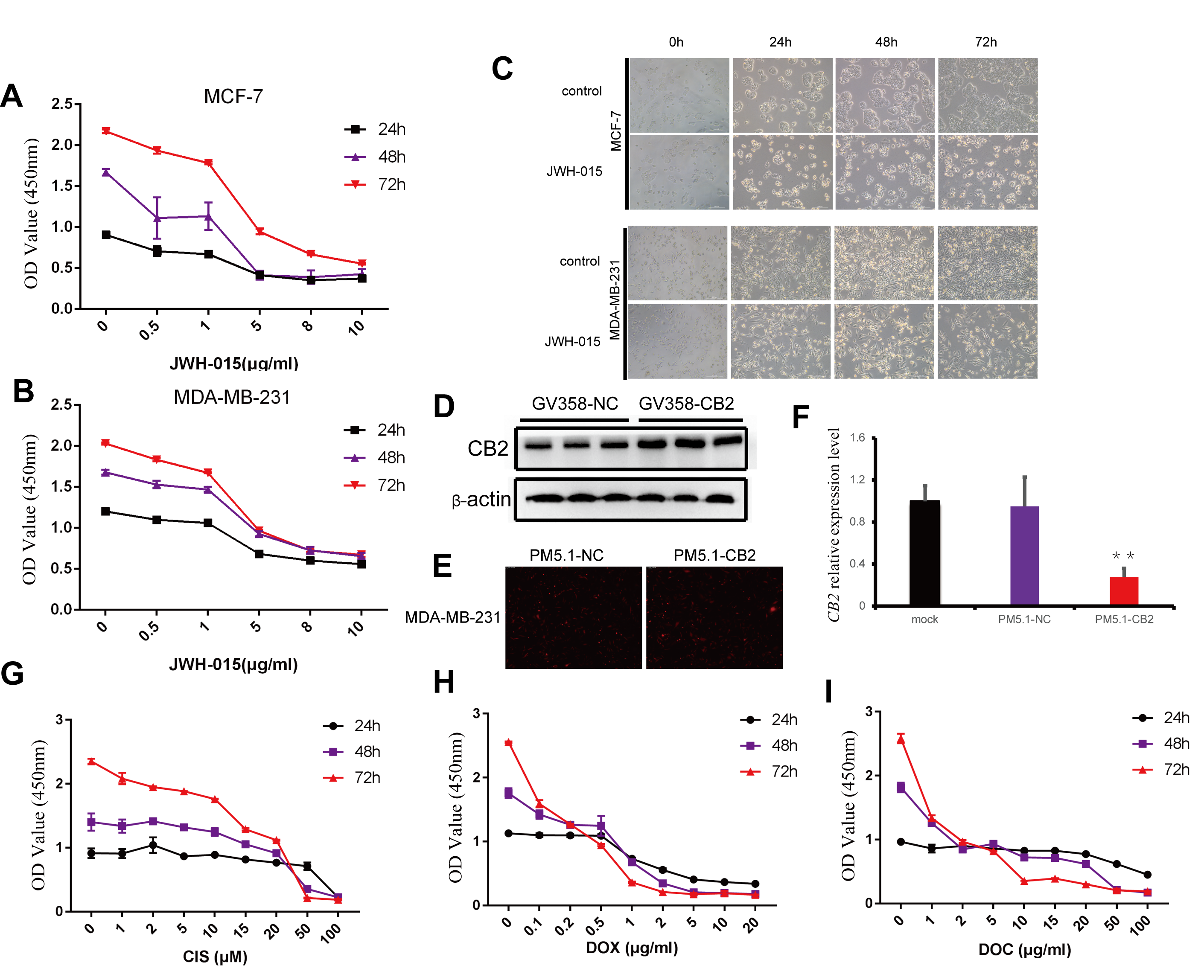

Supplement: Supplementary file 4 — Figure S4. [file CAM4-12-13538-s005.tif]
