# Supplementary material for: Overexpression of cannabinoid receptor 2 is associated with human breast cancer proliferation, apoptosis, chemosensitivity and prognosis via the PI3K/Akt/mTOR signaling pathway
Source: Cancer Med. 2023 May 23;12(12):13538–50. doi: 10.1002/cam4.6037 (PMC10315729; doi:10.1002/cam4.6037)
Supplement: Supplementary file 6 — Supplementary Figure Captions [file CAM4-12-13538-s001.docx]

**Figure S1.** Expression of *CB2* analyzed by immunohistochemical analysis. (A) The extent of immunohistochemical staining for *CB2* in breast cancer tissues, graded as 0-4. Grade 0 indicated no expression, grade 1–2 indicated low expression and grades 3-4 were defined as high expression. (B) Protein expression of CB2 and densitometric quantification.(C) Breast cancer patients with positive and negative *CB2* expression. (C) Expression of *CB2* in BC and adjacent non-cancerous tissues. Black arrows represent the para-cancer tissue, red arrows represent cancer tissue. Scale bars: 500 µm, 40×; 50 µm, 400×.

**Figure S2.** CB2 expression validation in different types of breast cancer from 95 patients.

**Figure S3.** The cell cycle changes afte overexpression of CB2 in MDA-MB-231 cell.

**Figure S4.** Effects of JWH-015, CIS, DOX and DOC on cell proliferation of MCF-7 and MDA-MB-231 cells. (A, B) The cell proliferation was analysed 24h, 48h and 72h after treated with different concentrations JWH-015 by CCK-8 assay in MCF-7 and MDA-MB-231cells, respectively. (C) Growth of BC cells treated with JWH-015 (5μg/ml) at 24h, 48h and 72h. (D) CB2 expression in the xenografts by western blot. (E)Red fluorescence was observed in MDA-MB-231 cell transfected with CB2-knockdown plasmid. Scale bar: 200 µm, 100×. (F) *CB2* mRNA expression level was determined in MDA-MB-231 cell CB2-after knockdown by qRT-PCR. (G-I) The cell proliferation was analysed 24h, 48h and 72h after treated with different concentrations CIS, DOX and DOC by CCK-8 assay in MDA-MB-231cells, respectively.
